# Editorial overview: From farms and forests to forks? A review of diagnosis and management of globally important zoonotic *Echinococcus* spp. cestodes

**DOI:** 10.1016/j.fawpar.2019.e00061

**Published:** 2019-06-27

**Authors:** Caroline F. Frey, Emily Jenkins, Britta Lundström-Stadelmann

**Affiliations:** a Centre for Food-borne and Animal Parasitology, Canadian Food Inspection Agency, Saskatoon, Canada; b Department of Veterinary Microbiology, Western College of Veterinary Medicine, University of Saskatchewan, Saskatoon, Canada; c Institute of Parasitology, Department of Infectious Diseases and Pathobiology, Vetsuisse Faculty, University of Bern, Bern, Switzerland

**Keywords:** *Echinococcus granulosus* s.l., *E. multilocularis*, Emergence, Diagnosis, Therapy, Control

## Abstract

This FAWPAR Special Issue is dedicated to zoonotic *Echinococcus* species. It is a compilation of invited papers that spans important aspects from molecular markers of emergence, diagnostics in both definitive and intermediate hosts, treatment of human alveolar echinococcosis, to control strategies in definitive hosts.

## Highlights

• Special Issue with invited manuscripts on zoonotic *Echinococcus* spp. (mainly *E. multilocularis*)
• Molecular markers of emergence help detect and date range expansions and introductions.
• Diagnostic advances in definitive and intermediate hosts that can improve surveillance
• Evidence based treatment of human alveolar echinococcosis
• Promising control strategies in definitive hosts to reduce human risk of exposure

## Graphical abstract

Topics of the Special Issue depicted on a scanning electron microscope image of the scolex of an adult *Echinococcus multilocularis*. Photo credit: Prof. Dr. A. Hemphill, Institute of Parasitology, University of Bern, Switzerland.

**Figure:**
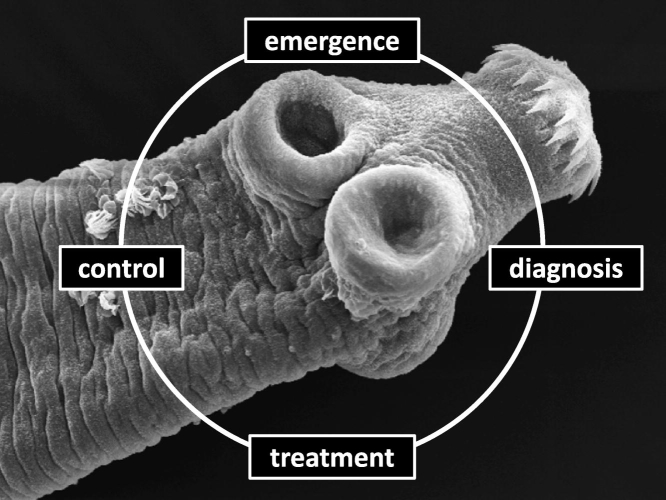

## 1 Introduction

This Special Issue (SI) of Food and Waterborne Parasitology focusses on zoonotic *Echinococcus* spp. mainly on *E. multilocularis*. The invited papers can be grouped into four main topics: molecular markers of emergence, diagnostics in both definitive and intermediate hosts, treatment of human alveolar echinococcosis (AE), and control strategies in definitive hosts (Table 1).

**Table 1:** Invited manuscripts in this Special Issue of Food and Waterborne Parasitology on zoonotic *Echinococcus* spp.

Section	Title	Authors	Weblink
Molecular markers of emergence	Genetic diversity of *Echinococcus multilocularis* in red foxes from two Scandinavian countries: Denmark and Sweden	Knapp J, Umhang G, Wahlström H, Al-Sabi MNS, Ågren EO, Enemark HL	https://doi.org/10.1016/j.fawpar.2019.e00045
Diagnosis in definitive and intermediate hosts	Validation of PCR-based protocols for the detection of *Echinococcus multilocularis* DNA in the final host using the Intestinal Scraping Technique as a reference	Maksimov P, Isaksson M, Schares G, Romig T, Conraths FJ	https://doi.org/10.1016/j.fawpar.2019.e00044
	A One Health systematic review of diagnostic tools for *Echinococcus multilocularis* surveillance: Towards equity in global detection	Schurer JM, Nishimwe A, Hakizimana D, Li H, Huang Y, Musabyimana JP, Tuyishime E, MacDonald LE	https://doi.org/10.1016/j.fawpar.2019.e00048
	Diagnostic and follow-up performance of serological tests for different forms/courses of alveolar echinococcosis	Gottstein B, Lachenmayer A, Beldi G, Wang J, Merkle B, Vu XL, Kurath U, Müller N	https://doi.org/10.1016/j.fawpar.2019.e00055
Treatment of human AE	The importance of being parasiticidal… an update on drug development for the treatment of alveolar echinococcosis	Lundström-Stadelmann B, Rufener R, Ritler D, Zurbriggen R, Hemphill A	https://doi.org/10.1016/j.fawpar.2019.e00040
	Surgical treatment strategies for hepatic alveolar echinococcosis	Salm LA, Lachenmayer A, Perrodin SF, Candinas D, Beldi G	https://doi.org/10.1016/j.fawpar.2019.e00050
	Elevated incidence of alveolar echinococcosis in immunocompromised patients	Lachenmayer A, Gebbers D, Gottstein B, Candinas D, Beldi G	https://doi.org/10.1016/j.fawpar.2019.e00060
	Surgery *versus* conservative drug therapy in alveolar echinococcosis patients in Germany – a health-related quality of life comparison	Schmidberger J, Steinbach J, Schlingeloff P, Kratzer W, Grüner B	https://doi.org/10.1016/j.fawpar.2019.e00057
Control in definitive hosts	Praziquantel treatment of dogs for four consecutive years decreased the transmission of *Echinococcus intermedius* G7 to pigs in villages in Lithuania	Šarkūnas M, Vienažindienė Z, Alvarez Rojas CA, Radziulis K, Deplazes P	https://doi.org/10.1016/j.fawpar.2019.e00043
	A systematic review and meta-analysis on anthelmintic control programs for *Echinococcus multilocularis* in wild and domestic carnivores	Umhang G, Possenti A, Colamesta V, d'Aguanno S, LaTorre G, Boué F, Casulli A	https://doi.org/10.1016/j.fawpar.2019.e00042

AE = alveolar echinococcosis.

*E. granulosus* sensu lato, the causative agent of cystic echinococcosis (CE), is a species complex with a predator – prey life cycle mainly involving canids and herbivores, although host assemblages vary according to parasite species (Thompson, 2017). Control of *E. granulosus* s.l. often depends on abattoir inspection and regulations to prevent feeding of offal to dogs, thereby breaking the domestic cycle of those parasites. In contrast, *E. multilocularis*, commonly known as the small fox tapeworm, cycles in wildlife and causes the disease AE in intermediate hosts. Its final hosts are mainly foxes and other canids, while wild rodents serve as natural intermediate hosts (Thompson, 2017). Humans, aberrant intermediate hosts for *Echinococcus* spp., can only become infected by oral ingestion of eggs shed by the final hosts. Therefore, human infections with *E. granulosus* s.l. and *E. multilocularis* are considered to be foodborne parasitic diseases, although waterborne transmission is also possible through eggs potentially present in unfiltered surface water. Due to their detrimental impact on human health, risks associated with both parasites regularly rank very high in comparison with other foodborne parasites (FAO/WHO, 2014; Bouwknegt et al., 2018).

## 2 Molecular markers of emergence

Alveolar echinococcosis is restricted to and a major concern in the northern hemisphere (Deplazes et al., 2017), where recent expansion of the historically endemic regions can be observed (Gottstein et al., 2015; Bebezov et al., 2018; Kotwa et al., 2019). This expansion has been documented by the finding of adult worms or their eggs in the canid final hosts, or by identifying the parasite in intermediate or aberrant hosts in novel regions. In North America, for example, pet dogs suffering from AE served as sentinels of range expansion (Jenkins et al., 2012). In contrast, surveillance in wild canids revealed newly endemic regions in Denmark and Sweden. In this special issue, Knapp and colleagues genotyped Scandinavian isolates of *E. multilocularis* using the microsatellite marker EmsB. The observed low genetic variation in the Scandinavian isolates compared to a collection of European isolates from seven endemic countries supports the relatively recent spread of *E. multilocularis* to Sweden and Denmark (Knapp et al., 2019). Furthermore, it highlights the usefulness of genotyping to better understand population dynamics in a seemingly uniform parasite based on morphology and sequencing of conserved regions of nuclear DNA.

## 3 Diagnosis in final and intermediate hosts

A central pillar in the control of zoonotic *Echinococcus* species is the diagnosis of infection in both definitive and intermediate hosts. While the gold standard in definitive hosts still is the recovery of adult worms, it is labor intensive, invasive and usually involves the death of the canid host (or uncomfortable purgation methods). Newer approaches using DNA extracted from fecal samples and various PCR protocols seem to achieve sensitivities and specificities that are comparable to the gold standard of the intestinal scraping technique, at least for *E. multilocularis*, as Maksimov et al. show in their thorough study (Maksimov et al., 2019). The possibility to screen fecal samples for the presence of *Echinococcus* spp. DNA will certainly facilitate more widespread and less invasive epidemiological investigations.

In their excellent One Health systematic review, Schurer et al. compile a treasure of information on diagnostic tools for *E. multilocularis* published in the last decade in humans, animals, and the environment (Schurer et al., 2019). Importantly, the authors also included papers originally published in Chinese – a source that is not always accessible for “western” researchers. China is the country with the most human AE cases and therefore it is important to consider original research from this vast region in any comprehensive review; unfortunately, due to the language barrier, there are few reviews incorporating this region, and we are proud to present one in this SI. The authors stressed that harmonization and collaboration across political boundaries is needed to achieve a comprehensive picture of the global situation of *E. multilocularis* and AE. One possible approach might be a global reference centre for zoonotic *Echinococcus* species, and global phylogeographic studies to better understand the significance of genetic diversity for ecology, epidemiology, and pathogenesis.

For the diagnosis of AE in humans, serology plays an important role along with imaging and histological and molecular techniques. We are happy to include in this SI an extensive review on the available serological tools in a leading diagnostic centre for AE. Gottstein and colleagues summarize the possibilities that serology offers for the diagnosis of human AE, and also give insights on how the antibody response changes over time after successful therapy (Gottstein et al., 2019). Thus, serology has merit not only for initial diagnosis, but also as an important and relatively inexpensive follow-up tool that provides clinicians with valuable information on disease regression or progression.

## 4 Treatment of human AE

Treatment of AE is based on surgical interventions combined with chemotherapy with benzimidazoles, or chemotherapy alone. Lundström-Stadelmann and colleagues demonstrate the search for additional drugs that might overcome the major drawback of benzimidazoles: they do not kill the parasite (*i.e.* they are parasitostatic), and thus therapy is often life-long and can have considerable drawbacks. In their excellent review they summarize efforts to discover new, parasiticidal drugs by repurposing old drugs (Lundström-Stadelmann et al., 2019). Quite uniquely in the world of cestode research, Lundström-Stadelmann et al. have established an *in vitro* system that allows high-throughput screening of drugs and compounds. We are also honoured to offer the insight of experienced surgeons at a Swiss referral centre for AE. Salm et al. share their valuable expertise on surgical treatment of human AE while also providing an overview of the respective literature (Salm et al., 2019). From the same group originates the study on the elevated incidence of AE in immunocompromised patients, underlining the importance of an intact immune system to fight against *E. multilocularis* (Lachenmayer et al., 2019). Last but not least, Schmidberger and colleagues from a German referral centre for AE assess the quality of life of AE patients after either surgery or conservative drug therapy, and found no significant differences between surgically or conservatively treated patients (Schmidberger et al., 2019). This may have important implications for low resource environments where treatment options may be restricted.

## 5 Control in definitive hosts

Definitive hosts for *Echinococcus* spp. are wild and domestic carnivores that shed infective eggs in their feces. Those eggs are the sole route of infection for humans and other animals acting as intermediate or aberrant hosts (Thompson, 2017). It is therefore intriguing to control infection in final hosts in order to prevent human infections or infection of livestock and pets. The study by Šarkūnas et al. included in this SI illustrates how local eradication of *E. intermedius* (*E. granulosus* G7) can be achieved by treating dogs every two months with praziquantel (Šarkūnas et al., 2019). The success of this intervention was facilitated by the unique characteristics of the village setting in Lithuania – with domestic dogs as the only final hosts, domestic pigs as intermediate hosts, and traditional slaughtering seasons during which transmission occurs from pigs to dogs over a circumscribed time period. This success story demonstrates that, despite the probable presence of a sylvatic cycle of G7 in wolves and wild boar in Lithuania, a targeted intervention in domestic animals can significantly reduce infection pressure for pigs, and probably also for humans. In the case of *E. multilocularis*, where the sylvatic cycle dominates, interventions to reduce prevalence tend to be much more complex. The systematic review by Umhang and colleagues on anthelmintic control programs in carnivores in endemic countries finds that monthly baiting of foxes with praziquantel drastically decreases the prevalence of this parasite in a short time period (Umhang et al., 2019). However, prevalence tends to rise again to pre-intervention levels when the baiting is stopped. The authors therefore correctly remind us that each intervention needs to have a favorable cost/benefit profile before it will be widely embraced by stakeholders. Nevertheless, under some ecological circumstances (for example, on islands) such interventions can profoundly reduce prevalence in intermediate hosts and probably human risk of exposure (Rausch et al., 1990).

## 6 Conclusions

The guest editors for this SI come from Canada and Switzerland, both regions with a high prevalence of *E. multilocularis* (and *E. canadensis* in Canada) in wildlife and in the fortunate situation of being wealthy countries. The contributions to this SI reflect this to a certain extent: most studies have been conducted in western countries, and most of the studies in the SI focus on *E. multilocularis*. Nevertheless, the guest editors are very much aware that echinococcosis (especially CE) is a major concern in many developing and resource-poor regions of the world. Some diagnostic and treatment options presented in manuscripts of this SI may not be realistic in more remote regions. However, it is our hope the innovations and new developments achieved under favorable economic and logistic circumstances will eventually be adapted to meet the needs of more remote or resource poor regions, and therefore help to advance the fight against these neglected parasitic diseases. The proposed global reference centre for zoonotic *Echinococcus* species might be a step in the right direction, which could facilitate standardization of diagnostic testing, curate evidence-based best practices for management of human cases and control of animal cycles, and coordinate studies taking a One Health approach to these parasites in people, pets, livestock, wildlife, and the environment.

## Declaration of Competing Interest

On behalf of all co-authors, I declare that there are no conflicts of interest regarding this submission.
